# Degradation and Extraction of Organochlorine Pollutants from Environmental Solids under Subcritical Water Conditions

**DOI:** 10.3390/molecules28145445

**Published:** 2023-07-16

**Authors:** Aaryn D. Jones, Andrew T. Morehead, Yu Yang

**Affiliations:** Department of Chemistry, East Carolina University, Greenville, NC 27858, USA

**Keywords:** subcritical water, oxidation, extraction, phenols, chlorophenols, organochlorine pesticides, contaminated soil

## Abstract

A subcritical water degradation and extraction method was developed to remediate environmental soils contaminated by highly recalcitrant organochlorine pollutants. Hydrogen peroxide was used to effectively decompose organochlorine pollutants under subcritical water conditions. As a method optimization study, the static wet oxidation of chlorophenols was first performed in subcritical water with and without added hydrogen peroxide. Complete oxidation was achieved using an added oxidant, and thus, the oxidation and extraction of chlorophenols from a sand matrix was then attempted. Complete oxidation and extraction with added oxidant were achieved within 30 min at 100 °C. We then investigated the subcritical water degradation and extraction of dieldrin, mirex, and p,p′-DDD. These organochlorine pesticides were not as easily oxidized as the chlorophenols, and the benefit of adding hydrogen peroxide was only clearly observed at 200 °C. Approximately a 20% increase in degradation was noted for each pesticide and insecticide at this temperature. Unfortunately, this difference was not observed with an increase in temperature to 250 °C, except in some cases, where the amount of degradation byproducts was reduced. Dieldrin and p,p′-DDD were essentially destroyed at 250 °C, while all the pesticides and the insecticides were completely removed from the sand at this temperature. The proposed method was then used to remediate a soil sample highly contaminated with DDT. The soil was obtained from the grounds of an old DDT mixing facility in Virginia and has been aging for several decades. Not only was 100% removal of DDT from this soil achieved using the proposed method at 250 °C, but also, the extracted DDT was completely destroyed during the process. The proposed remediation method, therefore, demonstrates a high potential as an efficient and environmentally sound technique for the detoxification of soils.

## 1. Introduction

### 1.1. Chlorinated Pollutants

The Environmental Protection Agency (EPA) has compiled a list of persistent, bioaccummulative, and toxic pollutants (PBTs) that pose a threat to both humans and natural ecosystems [1]. One class of compounds on this PBT list that are especially problematic within the soil systems are organochlorine pesticides and insecticides. Chlorinated pesticides, such as DDT, are known for their affinity for the nonpolar soil substrate rather than the polar water column in natural ecosystems. They are therefore quite persistent within the soil and are nearly inert to natural, physical, or chemical degradation, in some cases posing a threat to the integrity of the ecosystem for up to thirty years or more [1,2]. If exposed to mammals, these pollutants are stored and accumulated in the fatty tissues of the organism and will bio-magnify through the food chain. Many of these pollutants are proven or possible carcinogens, and well-documented evidence of the deleterious effects on wildlife has been shown. Due to the combination of these harmful properties, EPA has banned the use of all pollutants found on the PBT list. Although these pesticides are no longer approved for use, many problem areas still exist due to their environmental persistence. Therefore, attempts at soil remediation by removal of the harmful pollutants from the solid matrix should be taken in order to improve the quality of these disturbed ecosystems.

### 1.2. Traditional Remediation Methods

Typically, contaminated soils are dealt with by containment or incineration. Containment is simply the removal of the polluted soil and subsequent storage at a monitored hazardous waste site. Containment, however, may pose an eventual threat to the surrounding groundwater and requires long-term monitoring. Incineration is the complete combustion of the polluted soils at temperatures of about 1200 °C. This unfortunately creates a secondary pollution hazard arising from the emissions of the incinerator [3]. Additionally, both of the aforementioned methods are not ideal because they do not allow for the replacement of the soil to the original site.

A popular in situ method of treating polluted soils is bioremediation. This technique attempts to facilitate the biological breakdown of harmful organic pollutants by the available soil microorganisms. In most cases, the ground is tilled or injected with air to maintain the oxygen level. Also, in most cases, the appropriate microorganisms are not naturally present within the soils and must be artificially introduced into the soil.

Although widely sought as an in-situ pollution mitigation method, bioremediation has many disadvantages. The concentration of pollutants is critical to the survival of microorganisms, as a high concentration can be toxic while a low concentration can limit the biological activity of microorganisms. Uncontrollable variations in the environmental conditions at the site such as the natural flux in temperature, amount of sunlight, and frequency of precipitation can also impact microbial growth. Time is also likely to be a disadvantage, as bioremediation can take up to three years or more to completely remove toxins from a polluted site. During bioremediation, the monitoring and maintenance of the site is necessary to measure progress and optimize conditions, which can be quite costly and time consuming. Due to these disadvantages, alternate remediation methods such as extraction and chemical conversion techniques have been explored.

### 1.3. Extractions of Contaminated Environmental Solids

Traditional laboratory-scale methods of organic pollutant removal from environmental solids include Soxhlet extraction, liquid–solid extraction, and sonication. These methods not only necessitate the use of large amounts of organic solvents, but they are also time consuming, labor intensive, and essentially not suitable for the remediation of large volumes of polluted environmental solids. The use of organic solvents as an extraction media for polluted soil systems is not optimal because the organic solvents are often harmful themselves and solvent residues can remain on the soil after extraction. The need for cleaner extraction fluids has led to the use of sub- and supercritical fluids as extraction media.

Supercritical fluid extraction (SFE) typically makes use of supercritical carbon dioxide as the extraction fluid. SFE has been used at the industrial scale to extract caffeine from coffee beans and is a promising solvent for many other food preparation techniques due to the non-toxicity of carbon dioxide. Carbon dioxide is very cheap; however, the disadvantage is that the necessary instrumentation for SFE has a very high initial cost. In addition, due to the nonpolar nature of carbon dioxide, the extraction of more polar compounds such as organochlorine pesticides cannot be achieved efficiently without the addition of a small amount of organic modifier. 

This once again leads to the need for a clean extraction fluid for soil remediation purposes that can remove both polar and moderately polar pollutants. A solution to this problem has been shown to be the use of subcritical water as an extraction media. Subcritical water is simply heated water that is pressurized to maintain the liquid state. Under these conditions, subcritical water can mimic the physical and chemical properties of commonly used organic solvents, without the hazards associated with these undesirable solvents. Subcritical water has been proven to be a successful extraction fluid at the pilot-scale level for many pollutants commonly found in soils [4,5].

### 1.4. Chemical Conversion and Destruction Methods

As mentioned earlier, bioremediation is a popular method used to mitigate soil pollution via the biological transformation of the pollutants. Other methods are currently being studied, such as the chemical alteration of pollutants into innocuous or less persistent byproducts. These environmentally beneficial chemical reactions can be classified as oxidation, reduction, hydrolysis, or dechlorination.

Many types of reactions that facilitate the oxidation of persistent pollutants have been studied. Wet air oxidation has been frequently used to treat wastewater, which simply uses elevated temperatures and the oxygen available in the air to oxidize pollutants. For more persistent pollutants, however, stronger oxidizing agents must be added, such as ozone or hydrogen peroxide. Oxidation with hydrogen peroxide is of particular interest because it can be an extremely powerful oxidizing agent. The oxidative mechanism occurs due to the production of highly oxidizing hydroxyl radicals, which can be catalyzed by metals, UV light, and elevated temperature. Hydrogen peroxide oxidation with the addition of iron catalysts has been studied as an in-situ soil remediation method for gasoline-contaminated soils. Furthermore, the addition of hydrogen peroxide to wastewater or other environmental solids contaminated by polychlorinated biphenyls (PCBs) at elevated temperatures led to a significant reduction in the concentrations of these highly recalcitrant pollutants [6,7,8].

Chemical reactions can also be enhanced by using sub- or supercritical water as a reaction solvent. Supercritical water oxidation is a further development of the aforementioned wet air oxidation. Water in the supercritical state is combined with an oxidant to efficiently destroy pollutants in a matter of minutes. Supercritical water has been shown to effectively remediate soils contaminated with polycyclic aromatic hydrocarbons (PAHs) [9]. The supercritical water oxidation process has several disadvantages, however, which include equipment corrosion, salt deposition, and the high cost of the supercritical water oxidation apparatus.

To avoid the problems associated with supercritical water, a beneficial alternative could be the use of subcritical water as a reaction solvent. Many different types of reactions have been studied in subcritical water due to the relative ease of reaching the necessary conditions and the elimination of traditional organic solvents. Reactions such as oxidation, reduction, dechlorination, and hydrolysis have been performed in subcritical water [10,11,12,13,14,15]. Alkyl aromatic compounds were effectively oxidized to aldehydes, ketones, and acids using molecular oxygen mediated by transition metal catalysts in subcritical water. Several compounds such as methylene chloride and thiodiglycol have been observed to undergo hydrolysis and oxidation under subcritical water conditions [13]. The destruction of TNT in subcritical water was thought to proceed via a hydrolysis mechanism in a study by Hawthorne et. al., while in another study, aldehydes were reduced to alcohols with the addition of sodium formate under subcritical water conditions [5]. Reductive dechlorination of dioxins and pesticides has been performed in subcritical water both with and without the addition of zerovalent iron as a catalyst [14].

### 1.5. Organic Degradation Mechanisms

**Figure 1:**
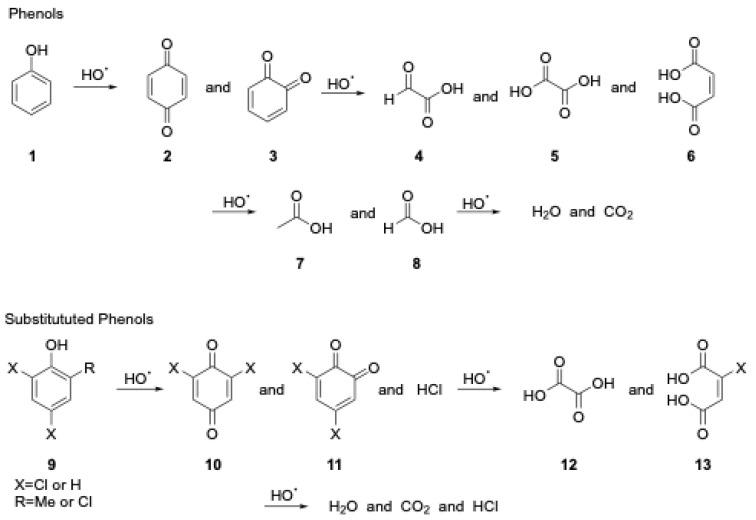
Generic mechanism for decomposition of phenols utilizing hydroxyl radicals.

The generic mechanism for the decomposition of phenols utilizing hydroxyl radicals (i.e., Fenton) proposed by Abouseoud et al. [15] is shown below in Figure 1. Other possible byproducts include acetic and formic acids. These reactions are most extensively studied under typical atmospheric pressure conditions and are most effective under acidic conditions [16]. The mechanism of phenols under subcritical water conditions is likely the same, but it is currently less clear how the different properties of the subcritical water and higher temperature and pressure will affect the outcome.

### 1.6. Purpose of This Study

Subcritical water has been shown to be an effective extraction fluid with success at the pilot scale thus far. Hydrogen peroxide oxidation has also been proven successful at destroying harmful pollutants with the creation of only innocuous byproducts, such as carbon dioxide and water. The purpose of this study is to combine the extraction efficiency of subcritical water with hydrogen peroxide oxidation in the hopes of completely remediating polluted soils while effectively destroying the removed pollutants. 

The wet oxidation of chlorophenols was performed with and without hydrogen peroxide to ensure that the oxidation reaction would occur under subcritical water conditions. The removal and destruction of chlorophenols, dieldrin, mirex, and p,p′-DDD from sand has been studied under subcritical water conditions with hydrogen peroxide as an added oxidant. A real-world soil sample contaminated by p,p′-DDT was oxidized and extracted using subcritical water under optimized conditions. This newly developed oxidation and extraction technique will hopefully lead to a clean remediation method capable of destroying and removing even the most recalcitrant organochlorine pesticides from environmental solids.

## 2. Results and Discussion

### 2.1. Wet Oxidation

Since a stoichiometric amount of hydrogen peroxide was available to oxidize all the compounds present in the vessels, it was hoped that the oxidation reaction would reach completion under subcritical water conditions. It was found that at 100 °C, 100% oxidation of o-cresol, 2-chlorophenol, and 94% oxidation of 2,3,6-trichlorophenol were achieved in those reactors that contained 3% hydrogen peroxide. No degradation occurred in the cells containing only water. It was thus assumed that phenols are readily degraded via an oxidative mechanism at 100 °C with the addition of an oxidant such as 3% hydrogen peroxide. These three compounds, however, do not appear susceptible to degradation under pure subcritical water conditions at 100 °C.

Another interesting fact was that no extraneous peaks were noted in the GC chromatograms. The absence of other peaks in the chromatograms means that oxidation byproducts were not isolated in the methylene chloride layer. This could mean that any oxidation byproducts were too polar to be extracted into the methylene chloride layer during the methylene chloride–water extraction, and thus would not appear in the GC chromatogram. It could also mean, however, that complete oxidation was achieved with only carbon dioxide, water, and hydrochloric acid as oxidation byproducts. This is quite plausible according to previous studies, which proved that phenol went through an unstable oxidation intermediate in subcritical water before it was completely oxidized to carbon dioxide and water under similar reaction conditions [17].

### 2.2. Spiking Studies

While the off-site spiking method (as discussed in Section 3) would have provided the best simulation of an actual environmental sample, it was found that a significant loss of spiked compounds (phenol, o-cresol, 2-chlorophenol, 2,4,-dichlorophenol, and 2,3,6-trichlorophenol) occurred during this process due to volatilization. However, there was no loss of the spiked compounds when top and gradual spiking methods were used.

In the traditional top spiking method, the compounds would be in a small, highly concentrated area. However, using the gradual spiking method, the compounds would be better dispersed throughout the solid matrix in the extraction cell. Since one of the goals of this study was to allow degradation reactions to occur under subcritical water conditions, it was felt that the compounds would be more likely to undergo uniform chemical conversion within the cell if they were more evenly dispersed. Therefore, with insignificant compound loss during the gradual spiking process, this method was chosen for all the subsequent experiments.

### 2.3. Oxidation and Extraction of Chlorophenols from Sand

In order to calculate the percent of pollutants extracted from the sand matrix during the experiment, Equation (1) was used. The percent removed reveals the extent to which the sand has been remediated:

% Removed = 100 − {(mass of pollutants in sand residue/total mass spiked) × 100}(1)

When calculating the percent of pollutants recovered from the extraction cell within the water extractant, Equation (2) was used. The percent recovered reveals the amount of pollutants that were extracted and recovered from the vessel but not degraded:

% Recovered = (mass of pollutants in water extractant/total mass spiked) × 100(2)

From both the amount remaining in the sand and the amount found in the water extractant, it was possible to calculate the extent of pollutant degraded based on the amount originally spiked into the extraction vessel. This relationship is shown in Equation (3), and it reveals the extent to which the originally spiked pollutants were oxidized (degraded) during the experiment:

% Degraded = 100 − % Residue − % Recovered(3)

Our first study focused on the effect of the static oxidation and extraction time on the percent of chlorophenols oxidized. Three different experiments were conducted at 100 °C with a 5, 20, and 60 min static oxidation and extraction step. All five phenols tested showed an increase in percent oxidation as the static extraction time increased. Note that the complete oxidization of all five compounds required 60 min.

It should also be noted that there were no phenols found in the methylene chloride layer after the sonication of the sand residue. Therefore, for all extraction conditions, 100% removal from the sand matrix was achieved for the phenols. There were also no extraneous peaks observed in GC chromatograms.

The next study focused on the effect of static oxidation and extraction temperature on the percent oxidation of the five phenols studied. Experiments were conducted with a one-hour static oxidation and extraction step at 25, 50, and 100 °C. All phenols tested showed an increase in oxidation efficiency as the temperature increased. At 25 °C, only 2–12% of the phenols was oxidized. The percentage of oxidized increased to 11–57% for phenol, o-cresol, and 2-chlorophenol at 50 °C while the oxidation and extraction efficiency for the di- and tri-chlorophenols stayed unchanged. However, all five phenols were completely oxidized at 100 °C.

In the kinetic study, there was a significant difference in the percent oxidized with a 5 min versus a 20 min static oxidation and extraction time. For example, with 5 min, none of the 2,3,6-trichlorophenol had been oxidized, while after 20 min, more than half of this compound (62%) had undergone oxidation.

It was also apparent that the lager chlorinated phenols were more difficult to be oxidized. In the 5 min experiment, 52% of the phenol had been oxidized, while the extent of oxidation was significantly less for mono-, di- and tri-chlorophenol, with 7, 2, and 0% oxidized, respectively. The kinetic study proved that there was a time effect on the oxidation efficiency. Each compound has a different response to the amount of time spent in the static oxidation and extraction step. As the number of chlorines in pesticides increases, longer static oxidation and extraction times are needed.

Similar trends were observed during the thermodynamic study, with the more chlorinated phenols being difficult to oxidize. When observing the trichlorophenol data, it was apparent that the compound needed a certain amount of activation energy to begin the oxidation process. This was assumed because at both 25 °C and 50 °C, there was essentially no trichlorophenol oxidation observed, while at 100 °C, the oxidation reaction had quickly reached completion. The other chlorinated phenols exhibited similar trends, with complete oxidation suddenly occurring at 100 °C. The thermodynamic study, therefore, made it obvious that a specific temperature must be reached for the oxidation reaction to occur to any significant extent for a specific compound. It also suggested that higher temperatures might be necessary to effectively oxidize compounds with a greater number of chlorine atoms.

### 2.4. Degradation and Extraction of PBT Compounds from Sand

#### 2.4.1. Dieldrin

The first dieldrin degradation and extraction experiment attempted was at 150 °C. Under this condition, the experiment was performed in triplicates both with and without hydrogen peroxide. As shown in Table 1, no increase in the degradation of dieldrin was achieved with added hydrogen peroxide.

Obviously, the destruction of dieldrin at this temperature was not aided by the addition of oxidant. Thus, the destruction of dieldrin that did occur was not likely to have reacted via an oxidative pathway. In a study by Kubatova et. al., only 5% of the chlorine in dieldrin was recovered as chloride ion under pure subcritical water conditions [18]. It was hypothesized that the hydrolysis of the epoxide ring of dieldrin had occurred, resulting in the formation of polar carboxylic acids that could not be identified by their methods. In this study, there were also no obvious byproducts present in the GC-FID chromatograms.

A higher temperature of 200 °C was attempted next in the hopes of further enhancing the destruction of dieldrin. As shown in Table 1, the destruction of dieldrin did increase at 200 °C when compared to the 150 °C experiment. The elevation of temperature resulted in an increase in the percent degraded for conditions with and without an oxidant. During this study, however, there was about a 20% increase in the percent of dieldrin degraded when an oxidant had been added to the extraction vessel. It was thus assumed that the temperature in this study was high enough to begin supplying sufficient energy to degrade dieldrin via an oxidative mechanism. 

A benefit to performing the experiment at 200 °C with the addition of hydrogen peroxide was that at this relatively low temperature, there was a significant amount (87%) of dieldrin degraded. There was a definite benefit to adding an oxidant at this temperature. Another benefit arising from performing the experiment at this temperature was that with a 98% removal of dieldrin from the sand, essentially all polluted sand had been remediated.

Yet, another increase in temperature was attempted in the hopes of completely destroying dieldrin. As shown in Table 1, the degradation and extraction of dieldrin at 250 °C was essentially complete. At 250 °C, there was sufficient energy to promote dieldrin destruction reactions in both pure subcritical water and with an added oxidant. 

In an attempt to find any polar reaction byproducts remaining in the water phase after the liquid–liquid extractions, UV–Vis analysis was performed on the water phases of the 250 °C experiment. A very large absorbance was found at about 200 nm for each water phase tested. This absorbance was at first attributed to the formation of polar byproducts that were possibly present within the water phase. Much later, however, two sets of blank extractions were performed, which proved that this absorbance was not due to dieldrin degradation. The first blank extraction was performed at 250 °C with un-spiked sand. The water phase of this extraction did not absorb UV light, which meant that the degradation and extraction system itself was not responsible for the UV peak at 200 nm. The second blank extraction, however, was also performed at 250 °C with sand that had been spiked with 10 μL of methylene chloride. This experiment was conducted because the solvent used to make all the pollutant spiking solutions was methylene chloride. The water effluent of this extraction resulted in the characteristic strong absorbance at 200 nm.

The identification of this UV-absorbing species was not extensively sought after; however, a study by Marrone et. al. suggested that methylene chloride undergoes hydrolysis in subcritical water. The hydrolysis byproducts formed in that study were formaldehyde and hydrochloric acid [19]. Formaldehyde may partially be responsible for this large absorbance at 200 nm. Also, the water phase of the second blank extraction was very acidic, with a pH approximately equal to 2. It can be assumed, therefore, that the dechlorination of methylene chloride occurs at 250 °C with the subsequent formation of a highly polar species that absorbs UV light at 200 nm.

**Figure 2:**
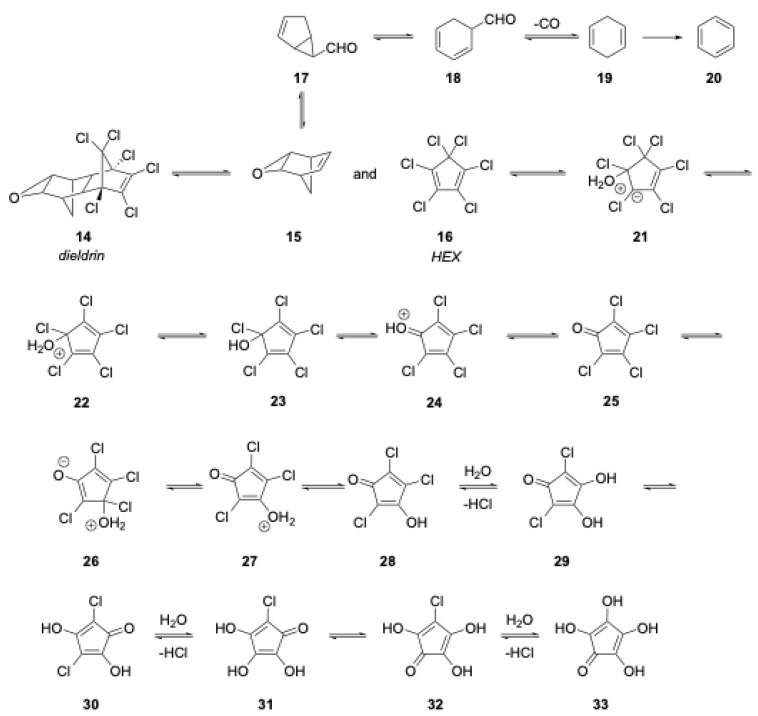
Proposed mechanism of degradation of dieldrin.

A proposed mechanism of degradation of dieldrin is shown below in Figure 2. It is very likely that the first critical step is a retro Diels–Alder reaction to result in norbornadiene oxide and hexachloropentadiene (HEX). Norbornadiene oxide can rearrange to benzene and CO, as shown [20]. At these temperatures, the addition of water to the highly reactive HEX should easily lead to tetrachlorocyclopentadienone [21], which can undergo a series of addition/elimination reactions with water (or hydroxyl radical) to form a series of highly polar (and almost certainly water soluble) compounds. The degradation of the product and its tautomers has been studied under acidic conditions [22].

#### 2.4.2. Mirex

**Figure 3:**
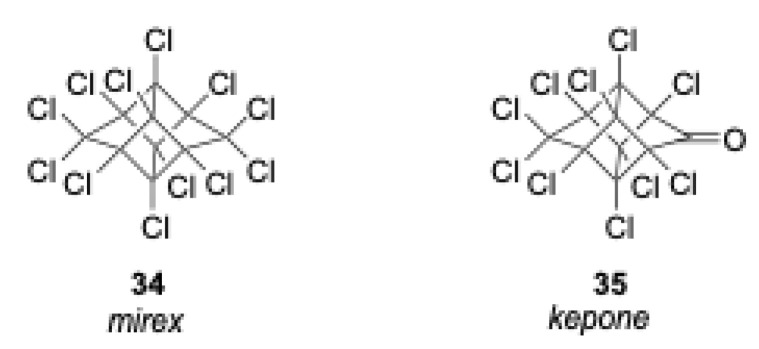
Structures of mirex (**left**) and kepone (**right**).

Mirex is an organochlorine insecticide that was used extensively in the southern United States to control the fire ant population during the years 1962–1978. The insecticide was banned in 1978 due to the discovery of its significant bioaccumulation and its high toxicity to marine crustaceans. This pesticide has also been clearly linked to carcinogenic activity. Mirex is known to be an extremely stable compound with a melting point of 485 °C. It is quite resistant to natural biological and chemical degradation, however, degrades extremely slowly into kepone and dechlorinated mirex derivatives. Mirex is also known to strongly adsorb onto the organic matter within soils, thus necessitating the remediation of problem areas. The destructive oxidation of mirex has been reported [23]. EPA has made mirex a priority pollutant due to its persistence in the environment and potential health risks. The structures of mirex and kepone are shown in Figure 3.

The first mirex degradation and extraction experiment attempted was performed at a temperature of 200 °C, both with and without 3% hydrogen peroxide. As shown in Table 2, the percent removed at 200 °C ranges from 91 to 99, while the percent degraded is 33 and 50 for experiments with pure water and 3% hydrogen peroxide, respectively. However, the percent removed at 250 °C reaches 100 for experiments with both pure water and 3% hydrogen peroxide, as shown in Table 2. The percent degraded also increases to 58 for experiments with both pure water and 3% hydrogen peroxide.

**Figure 4:**
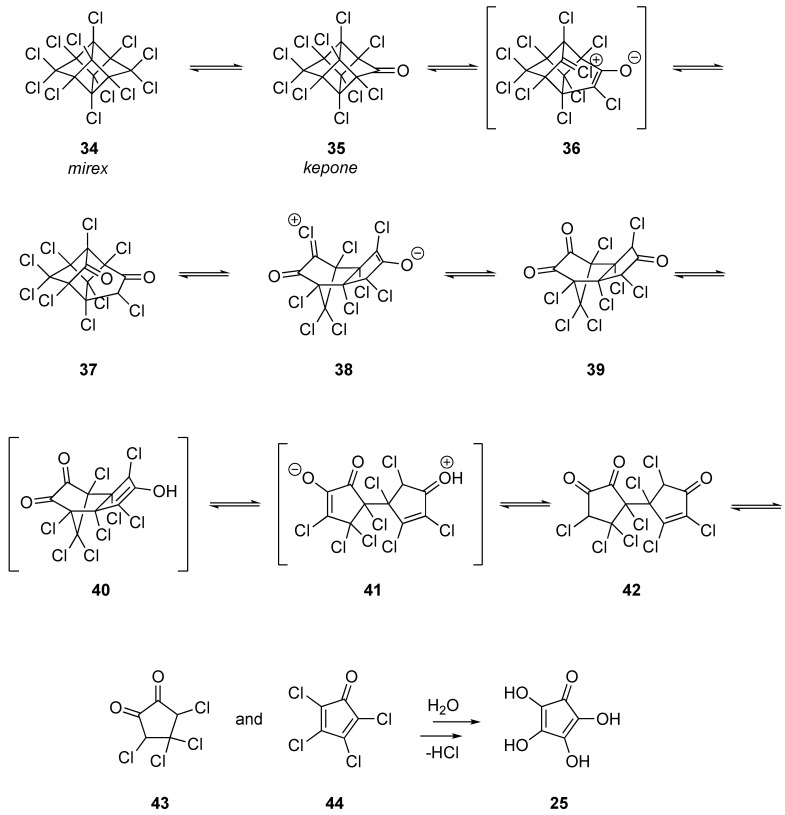
Mechanism of the degradation of mirex.

The mechanism of the degradation of mirex is well-established to begin with hydrolysis to kepone as shown in Figure 4 [24]. The cleavage of the highly strained four-membered rings can be considered to be analogous to a retro-aldol, ultimately resulting in the tricyclic norbornadiene with a fused cyclopentanone. Two additional retro-Michael reactions result in two monocyclic compounds that can undergo successive addition/elimination reactions of water (or hydroxyl radicals if present) to generate the tetrahydroxycyclopentadienone, the decomposition of which is referenced above for dieldrin.

#### 2.4.3. p,p′-DDD

**Figure 5:**
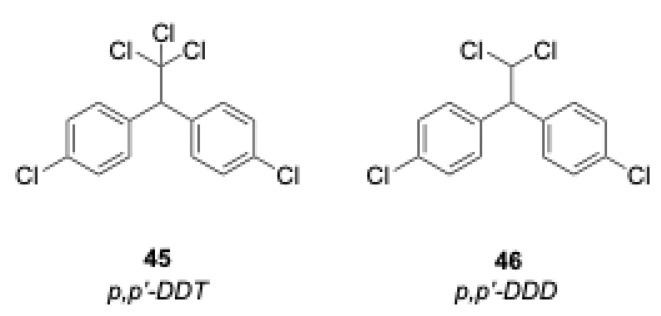
Structures of p,p′-DDT and p,p′-DDD.

DDT is perhaps the most well-known chlorinated pesticide due to the highly publicized discovery of the deleterious effects this compound has on wildlife in the late 1960s. Prior to 1972, this compound was the most commonly used pesticide in agricultural practices. The use of this pesticide in the United States was banned in 1972. DDT itself is not often as persistent in the environment as its dechlorinated byproducts, DDD and DDE [25]. EPA has targeted all three compounds as priority pollutants due to their persistence and potential health risks. These compounds are all probable carcinogens and bioaccumulate up the food chain. They also can persist up to 15 years within the soil before beginning to degrade under natural conditions. DDD was chosen for the next experiment because it is such a prevalent pollutant in areas previously contaminated by DDT. The structures of p,p′-DDT and p,p′-DDD are shown in Figure 5.

The first DDD degradation and extraction experiment attempted was performed at 200 °C. As shown in Table 3, the percent of DDD degraded was about 20% higher for those experiments with added hydrogen peroxide. Thus, at this temperature, there was a definite benefit to adding an oxidant to the extraction vessel.

An increase in temperature to 250 °C was attempted to determine if the complete degradation of DDD could be achieved. The results (Table 3) of this experiment showed that an identical near 100% removal was achieved for experiments with and without hydrogen peroxide. At this temperature, nearly 93% DDD was degraded for experiments with and without an added oxidant.

Each of the three pollutant studies had several common experimental results. For example, each pollutant exhibited similar degradation trends with degradation and extraction at 200 °C. The specific trend noted was a similar increase in the percent pollutant degradation during the experiments in which an oxidant had been added. For dieldrin, mirex, and DDD, the increase in percent degradation with an added oxidant was 22%, 16%, and 21%, respectively. There was an obvious advantage to adding an oxidant at this temperature for each pollutant.

An increase in temperature to 250 °C, however, served to close this gap between the amount of pollutant degradation found in those experiments with and without added oxidant. In fact, the percent difference between those experiments with and without added oxidant was very small. For diedrin, mirex, and DDD, the percent difference between the percent degradation in these two experiments at 250 °C was 2%, 0%, and 0%, respectively. An explanation of this occurrence could be that at 250 °C, the degradation reactions have sufficient energy to proceed in pure subcritical water without the benefit of an added oxidant. 

**Figure 6:**
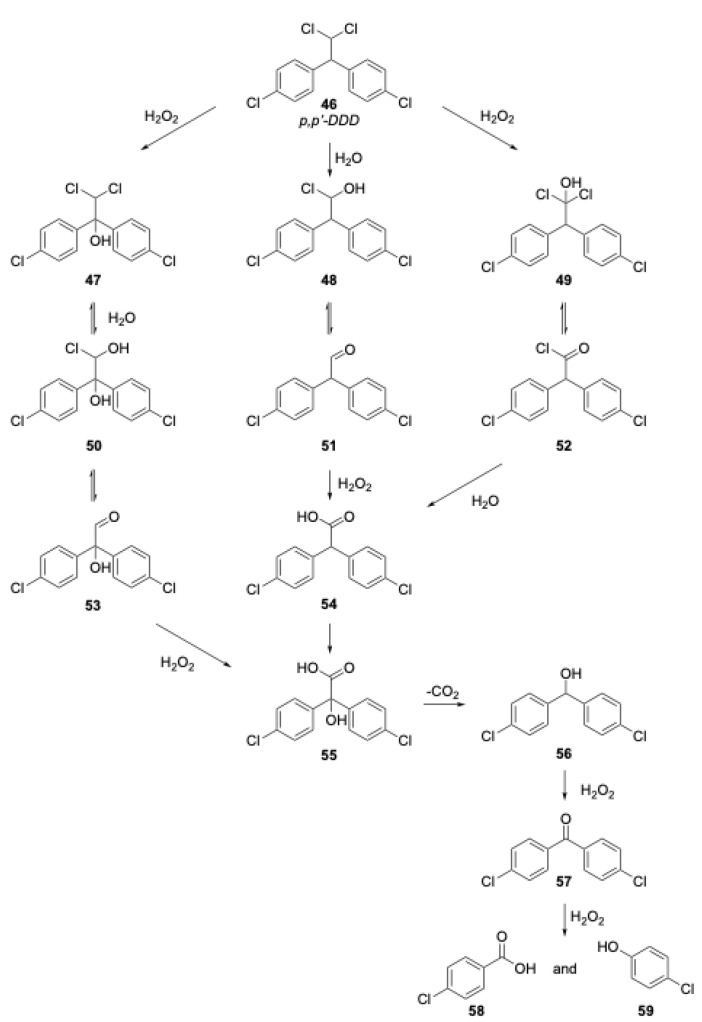
Proposed mechanism for the degradation of DDD.

The proposed mechanism for the degradation of DDD is shown below in Figure 6. The most likely pathway is the initial hydrolysis and formation of the aldehyde shown in the upper part of the figure. In the absence of an oxidant (or a limited amount of dissolved oxygen), it is likely that slow decarbonylation or the limited oxidation of that aldehyde will lead to a radical degradation pathway, implicating a number of the intermediates shown. The presence of added oxidant will likely speed the decomposition of many of those intermediates, ultimately leading to the p-chlorobenzoic acid and p-chlorophenol shown, which will degrade as discussed in the generic mechanism shown earlier. The degradation of DDT discussed below is likely to follow a similar pathway since a major product of decomposition of DDT is DDD.

### 2.5. Degradation and Extraction of DDT from Environmental Solid Samples

A soil sample known to be heavily contaminated with DDT was obtained from a contact within the Army Corps of Engineers. The soil was obtained from the John H. Kerr Dam and Reservoir, which is located in central Virginia on the North Carolina border. The Corps operates this reservoir and the 50,000 acres of surrounding land. Until the early 1970s, one area of this land housed a DDT mixing facility. The purpose of this facility was to mix DDT with fuel oil for use in mosquito vector control projects. Due to concern for human health, the site was sampled in December of 1998 at the request of EPA. The pollutants still found present within the soil were DDT, DDD, and DDE at maximum concentrations of 6000 mg/kg, 670 mg/kg, and 4600 mg/kg, respectively. Note that the soil used in this study has been contaminated and aged for decades.

The degradation and extraction of a pollutant from a real-world environmental solid sample, was attempted to determine the validity of the remediation process using only pure subcritical water. As shown in Table 4, 94% DDT was degraded at 200 °C. The percent degradation of DDT increased to 99.8% when the water temperature was increased to 250 °C. These results demonstrate that pure subcritical water is capable of efficiently decomposing DDT contained in real-world solid samples at 250 °C.

## 3. Experimental Section

### 3.1. Reagents and Materials

Phenol, *o*-cresol, menthol, 2-chlorophenol, 2,4-dichlorophenol, 2,3,6,-trichlorophenol and phenanthrene were purchased from Sigma-Aldrich, St. Louis, MO, USA. A total of 3% hydrogen peroxide, methylene chloride, Ottawa sand standard (20–30 mesh), *p*-xylene, sodium sulfate, acetone, and *n*-hexane were obtained from (Fisher Scientific, Fair Lawn, NJ, USA). Dieldrin, mirex, p,p′-DDT, and p,p′-DDD were acquired from AccuStandard (New Haven, CT, USA). Deionized water (18 MΩ-cm) was prepared in our laboratory.

A 6890 GC-FID was purchased from Hewlett-Packard (Palo Alto, CA, USA). A HP-35MS column (30 m × 0.25 mm inner diameter with a 0.25 μm film thickness) used for GC analysis of phenols and a HP-5 column (30 m × 0.32 mm inner diameter with a 0.25 μm film thickness) used for PBT analysis were both bought from Hewlett-Packard. A syringe pump (260 D) was purchased from ISCO (Lincoln, NE, USA). Stainless steel extraction vessels (50 mm × 4.6 mm) were acquired from Keystone Scientific (Bellefonte, PA, USA), and two-way stainless steel shut-off valves (HIP model 15-11AF1) were obtained from High Pressure Equipment (Erie, PA, USA). Stainless steel static extraction vessels (3.18 mL, 5 cm × 9 mm) were obtained from Raleigh Valve and Fitting Company (Raleigh, NC, USA). Isotemp ovens and sonicators were purchased from Fisher Scientific.

### 3.2. Standard and Sample Preparations

For wet oxidation experiments, the maximum concentration of each compound in the methylene chloride layer after the experiment could have been as follows: 2933 ppm for both 2-chlorophenol and *o*-cresol, and 1467 ppm for 2,3,6-trichlorophenol. Thus, four standard solutions were prepared in 1.5 mL of methylene chloride at 3000, 1500, 500, and 100 ppm for each compound. An amount of 10 μL of the 10,000 ppm menthol solution was then added to each standard solution to serve as an internal standard.

For spiking studies, the maximum concentration of each compound in the methylene chloride layer after the experiment could have been 200 ppm for each compound. Therefore, four standard solutions were prepared in 1.5 mL of methylene chloride at 400, 200, 20, and 5 ppm for each compound. An amount of 10 μL of the 30,000 ppm *p*-xylene solution was then added to each standard solution as an internal standard.

The maximum concentration of each compound in the methylene chloride layer after the chlorine phenols experiment could have been 20 ppm for each compound. Thus, four standard solutions were prepared in 1.5 mL of methylene chloride at 100, 50, 20, and 5 ppm for each compound. An amount of 10 μL of the 15,000 ppm *p*-xylene solution was then added to each standard solution.

Since the maximum concentration of any pollutant in the methylene chloride layer after the PBT experiments was estimated to be approximately 133 ppm, four standard solutions were prepared in 1.5 mL of methylene chloride at concentrations of 200, 100, 20, and 5 ppm. An amount of 10 μL of the 5000 ppm phenanthrene solution was then added to each standard solution to serve as an internal standard.

The maximum concentration of DDT in the methylene chloride layer after the experiment could have been approximately 290 ppm. Thus, four standard solutions were prepared in 1.5 mL of methylene chloride at concentrations of 400, 200, 100, and 50 ppm. Four standards of DDD were also prepared at 400, 200, 100, and 50 ppm. An amount of 10 μL of a 5000 ppm phenanthrene solution was then added to each standard solution to serve as an internal standard.

### 3.3. Procedures

#### 3.3.1. Wet Oxidation

An amount of 2.2 mg of 2,3,6,-trichlorophenol was weighed into a stainless steel static extraction vessel (3.18 mL, 5 cm × 9 mm). An amount of 3 mL of pure deionized water or 3% hydrogen peroxide solution was then added into the loaded vessels, with quadruplicate experiments being performed for both conditions. An amount of 4 μL of 2-chlorophenol and *o*-cresol were directly injected into the liquid present within the vessel. Each vessel was then tightly sealed and placed into a preheated Isotemp oven at 100 °C for one hour. After the desired reaction time, the vessels were removed from the oven and cooled to room temperature. A 10,000 ppm menthol solution was prepared in methylene chloride to serve as an internal standard. An amount of 10 μL of this solution was injected into the liquid inside each vessel. This was followed by the transfer of this liquid into a glass vial. Exactly 0.75 mL of methylene chloride was added to each empty vessel as a rinse, to ensure the quantitative removal of the compounds from the reaction vessel. This volume of methylene chloride was then removed from each vessel and added to the glass vial containing the aqueous layer from that vessel. A liquid–liquid (methylene chloride–water) extraction was performed, during which the vials were vigorously shaken for one minute. The methylene chloride layer was then transferred into another vial. Another 0.75 mL of methylene chloride was added to the water layer. The liquid–liquid extraction was repeated, the methylene chloride layer was once again removed and combined with the first fraction of methylene chloride for gas chromatographic (GC) analysis.

#### 3.3.2. Spiking Methods

Studies of pollutant extractions from a solid matrix were conducted using pure laboratory sand that had been spiked with the targeted pollutants. Initial studies on three different spiking methods were performed in order to determine which method would best simulate an actual contaminated solid sample.

Traditionally, most extractions of solids have been performed by first filling an extraction vessel with the solid (e.g., soils, sludges, or sediments), followed by spiking the compounds directly on the top of the solid. This method, however, was determined to be a poor simulation of a real contaminated environmental sample because the pollutants are not evenly distributed throughout the solid sample matrix.

An alternative to this traditional method was to openly spike a pre-weighed amount of sand with the pollutants, thus mixing them together prior to placement within the extraction vessel. A disadvantage to this off-site spiking approach is that the amount of compounds lost due to evaporation during the spiking and mixing process was unknown and needed to be investigated.

**Figure 7:**
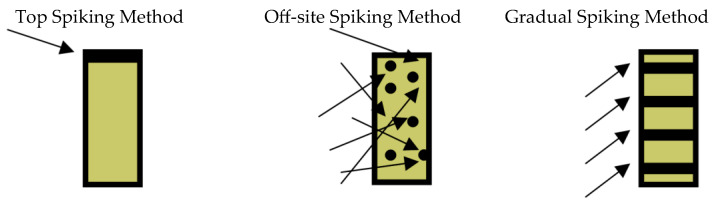
Pollutant distribution after each spiking method.

Yet, another approach was attempted in the event pollutant volatilization was found to be significant during the off-site spiking method. This approach, the gradual spiking method, was one in which the compounds were spiked as the extraction vessel was filled with sand. This method was investigated in the hopes of minimizing any potential volatilization, while more evenly distributing the pollutants throughout the vessel than the traditional top spiking method. Figure 7 is a visual representation of the pollutant distribution in the vessel after each spiking method.

A concentrated spiking solution containing 90 mg each of phenol, *o*-cresol, 2-chlorophenol, 2,4-dichlorophenol, and 2,3,6-trichlorophenol was prepared and diluted in 3 mL of methylene chloride. This resulted in a spiking solution that contained 30,000 ppm of each compound. 

Ottawa sand (20–30 mesh) was used as the solid extraction matrix. Prior to spiking, this sand was first cleaned with two methylene chloride rinses, followed by an acetone rinse. The sand was then dried in an oven at 100 °C for one hour.

Approximately 1.18 g of the pre-cleaned sand was spiked with 10 μL of the 30,000 ppm spiking solution for each study. For the top spiking method, which was investigated as a control, the sand was placed into a glass vial and spiked directly on the surface with the spiking solution. The vial was then immediately closed.

During the off-site spiking method, the sand was placed into a weighing boat for the spiking process. The spiking solution was applied as evenly as possible onto the sand and was mixed thoroughly with the needle of the spiking syringe. The spiked sand was then transferred to a glass vial and immediately closed.

For the gradual spiking method, approximately ¼ of the pre-weighed sand was added to the vial and 2.5 μL of spiking solution was added to the surface of this layer of sand. This process was repeated until there were four stratified layers of applied pollutants in the vial, with a total applied spiking solution volume of 10 μL.

In order to analyze the amount of pollutants remaining in the sand after each spiking method, 1.5 mL of methylene chloride was added to each vial. An amount of 10 μL of 30,000 ppm *p*-xylene solution was added to the methylene chloride phase in each vial, serving as an internal standard. All the vials were tightly wrapped with parafilm and sonicated in a sonicator for two hours.

#### 3.3.3. Oxidation and Extraction of Chlorophenols from Sand

Prior to attempting the oxidation and extraction of PBT pollutants from a solid matrix, the method was first optimized using chlorophenols. These compounds were chosen because they are relatively cheap and could serve as model compounds for the chlorinated pollutants on the PBT list. The thermodynamic and kinetic effects on oxidation and extraction efficiency were investigated in this study.

##### 3.3.3.1. Hydrogen Peroxide Oxidation and Subcritical Water Extraction System

**Figure 8:**
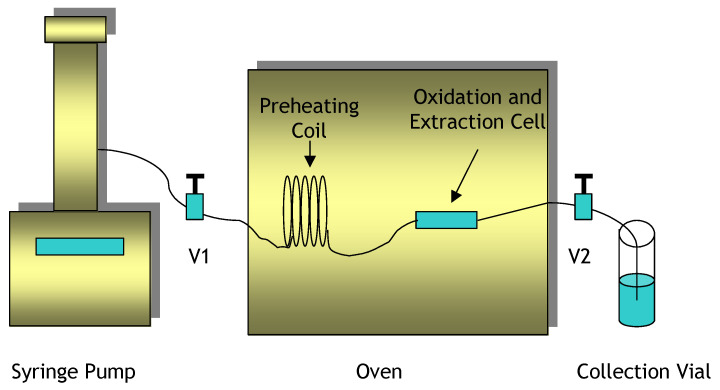
Subcritical water oxidation and extraction system.

The system shown in Figure 8 consists of an ISCO 260 D syringe pump, a Fisher Isotemp oven, a Keystone SFE dynamic extraction vessel (50 mm × 4.6 mm), and two two-way shut-off valves.

For each experiment, the oven was first turned on and set to the desired temperature. While the oven was equilibrating, the extraction vessel was prepared. One end of the vessel was tightly closed and then turned upright for the loading of sand according to the gradual spiking method. One fourth of the total amount of sand was added to the vessel followed by the application of 2.5 μL of spiking solution. An amount of 50 μL of 3% hydrogen peroxide was also applied to this first layer of sand. Then, another layer of sand was added, spiked with 2.5 μL of spiking solution and 50 μL of 3% hydrogen peroxide. This process was repeated twice more until four layers of sand spiked with spiking solution were contained within the extraction vessel. A total volume of 10 μL of spiking solution and 200 μL of 3% hydrogen peroxide had been added to the vessel. The other end of the vessel was tightly closed and then the vessel was connected to the oxidation and extraction system.

Once connected to the system, V2 was closed, and the vessel was pressurized to 15 atm by operating the syringe pump in the constant pressure mode. This step allowed a check for leaks in the system, while also supplying sufficient pressure to keep the water in the liquid state during the subsequent heating step. After finding no leaks in the system, V1 was closed, and the extraction vessel was placed inside the oven. A static oxidation and extraction step was performed for a specified amount of time with both V1 and V2 closed. At the end of the static step, V2 was opened, the pump was engaged in the constant flow mode at a flowrate of 0.5 mL/min, and V1 was opened. This dynamic extraction step was performed for 10 min, with 5 mL of the effluent water being collected in a vial. After 10 min, the extraction vessel was removed from the oven and the pump was turned off. After allowing the vessel to cool, it was disconnected from the oxidation and extraction system.

##### 3.3.3.2. Preparation for Analysis

**Figure 9:**
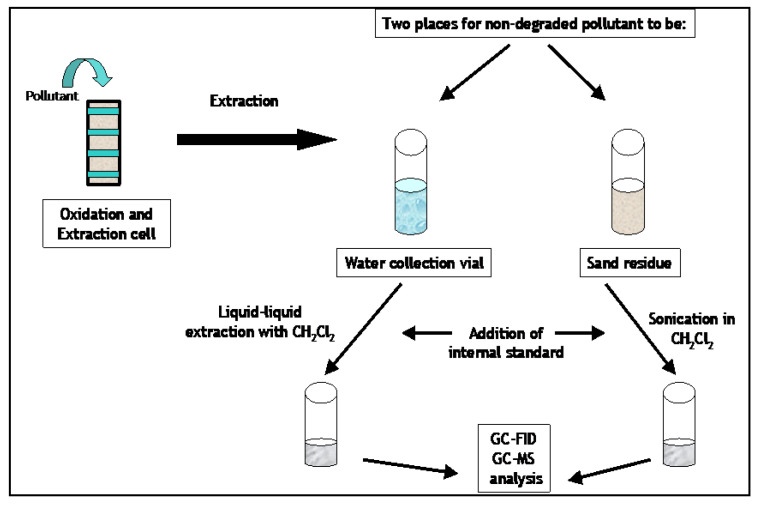
Schematic of analysis procedure.

Quantification of the amount of pollutants left after the oxidation and extraction processes were completed using the GC-FID. In preparation for GC analysis, the water effluent collected during the dynamic extraction step underwent a liquid–liquid extraction with methylene chloride, while the sand residue was sonicated in methylene chloride to remove residual pollutants. Figure 9 is a simple schematic of the analysis procedure that is described in further detail in the following paragraphs.

##### 3.3.3.3. Water Extract

A liquid–liquid extraction was performed to remove the extracted pollutants from the water phase for GC analysis. Exactly 0.75 mL of methylene chloride was added to the 5 mL of water effluent. A total volume of 10 μL of 15,000 ppm *p*-xylene solution was added to the methylene chloride layer as an internal standard. The vial was then vigorously shaken for exactly one minute. After the liquid phases were well-separated again, the methylene chloride layer was removed and placed into another vial. Another 0.75 mL of methylene chloride was then added to the water effluent for the second liquid–liquid extraction. The vial was shaken again for exactly one minute. The second methylene chloride layer was removed and placed into the vial with the first. The contents of this vial were injected into the GC to quantify the amount of pollutants that had been present within the water effluent.

##### 3.3.3.4. Sand Residues

After removing the extraction vessel from the system, the vessel was carefully opened. The sand inside the vessel was quantitatively transferred into a vial. Exactly 1.5 mL of methylene chloride was added to this vial, along with 10 μL of the 15,000 ppm *p*-xylene solution. The vial was tightly wrapped with parafilm to prevent evaporation and was then placed into a sonicator bath for four hours. Immediately after sonication, the contents of the vial were injected into the GC to quantify the amount of pollutants that had remained on the sand after the oxidation and extraction process.

### 3.4. Degradation and Extraction of PBT Pollutants from Sand

Following the optimization of chlorophenol oxidation and extraction from a solid matrix, the degradation and extraction of several of EPA’s PBT compounds from a solid matrix was attempted. Three different persistent and problematic pollutants were chosen from the PBT list for the experiment: dieldrin, mirex, and p,p′-DDD. A general procedure for the degradation and extraction of each pollutant was followed and is described in the next section. The results of the experiment for each individual pollutant will then be discussed separately.

The degradation and extraction instrument used in these studies is the same instrument described in Section 3.3.3.1 as shown in Figure 8. The analysis was carried out the same way as described in Section 3.3.3.2 and Figure 9. Quantification of the amount of pollutants left after the degradation and extraction processes was completed using the GC-FID. In preparation for GC analysis, the water effluent collected during the dynamic extraction step underwent a liquid–liquid extraction with methylene chloride, while the sand was sonicated in methylene chloride to remove residual pollutants. 

The water extract was treated and analyzed in the same way as described in Section 3.3.3.3 and the sand residue in Section 3.3.3.4. The dieldrin degradation and extraction experiments were performed at three different temperatures of 150, 200, and 250 °C. The length of the static degradation and extraction step, however, was constant for all the experiments with a duration of 20 min. All experiments under each of the six conditions were repeated in triplicate. DDT degradation and extraction from an environmental solid was attempted under four different conditions. The experiments were performed at temperatures of 200 °C and 250 °C both with and without added hydrogen peroxide. The length of the static degradation and extraction step was 45 min. All experiments were repeated in triplicate.

### 3.5. Degradation and Extraction of DDT from an Environmental Soil Sample

The degradation and extraction instrument used in these studies is the same instrument described in Section 3.3.3.1 as shown in Figure 8. The analysis was carried out the same way as described in Section 3.3.3.2 and Figure 9. The water extract was treated and analyzed in the same way as described in Section 3.3.3.3 and the sand residue in Section 3.3.3.4. DDT degradation and extraction from an environmental solid was attempted under four different conditions. The experiments were performed at temperatures of 200 °C and 250 °C both with and without added 3% hydrogen peroxide. The length of the static degradation and extraction step was 45 min. All experiments were repeated in triplicate.

### 3.6. Gas Chromatographic Analysis

A Hewlett-Packard 6890 GC-FID was used to analyze the methylene chloride layers from each extraction vessel. A total volume of 1.5 μL of each sample was injected into the GC. The injection mode was splitless and the injector temperature was 275 °C. The GC capillary column employed was a HP-35MS (30 m × 0.25 mm inner diameter with a 0.25 μm film thickness) for wet oxidation, spiking, and chlorophenol investigations and a HP-5 (30 m × 0.32 mm inner diameter with a 0.25 μm film thickness) for PBT and DDT experiments. The initial oven temperature was 80 °C with a 15 °C increase per minute until reaching the final temperature of 320 °C. This final temperature was held for five additional minutes. The flame ionization detector (FID) was operating at a temperature of 300 °C.

### 3.7. UV–Vis Analysis

In an attempt to determine if there were any polar byproducts present in the water phase after the liquid–liquid extraction, UV–Vis analysis of some water effluents was performed. The instrument employed was a Hewlett-Packard 8453 Ultraviolet–Visible Spectrophotometer. The samples were housed in a quartz cuvette (Fisher), and the absorption of the samples was scanned from 190 to 1100 nm.

## 4. Conclusions

The preliminary wet oxidation study proved that the complete oxidation of phenols could be achieved under subcritical water conditions with an added oxidant. Later, extractions of phenols from a sand matrix were also proven to be a success, with complete oxidation and extraction occurring in only 30 min at a temperature of 100 °C.

PBT pollutants dieldrin, mirex, and p,p′-DDD were also degraded and extracted from a sand matrix. These compounds were not as readily oxidized as the phenols, and the benefit of adding an oxidant was only clearly seen at 200 °C for each pollutant. At this temperature, the increase in the degradation of each pollutant was about 20% when oxidant was added. An increase in temperature to 250 °C enhanced the degradation strength of pure subcritical water, and thus, very little difference in degradation was noted between those experiments with and without added oxidant. However, there was still a benefit to the addition of an oxidant at this temperature when considering the degradation of DDD and the subsequent formation of degradation byproducts. The amount of these byproducts remaining in the water effluent was clearly lower after the experiments with added oxidant, thus creating a cleaner extract.

The final goal of the project was to attempt the degradation and extraction of DDT and DDD from a highly contaminated environmental soil sample. The concentration of DDT in the soil was significantly reduced at a temperature of 200 °C; however, the formation of a byproduct (DDD) was noted. Fortunately, the amount of DDD formed as a byproduct was greatly reduced in experiments that employed an oxidant. With an increase in temperature to 250 °C, DDT remediation was complete in both systems, and DDD was not formed as a DDT degradation byproduct. In fact, the original concentration of DDD in the soil was reduced at this temperature; thus, the remediation of both DDT and DDD from the soil was achieved under these conditions. The potential of the proposed remediation technique in the real world was shown on the laboratory scale with the completion of this study.

The potential application of this remediation technique would most likely apply to relatively small, yet highly contaminated sites for which fast remediation was desired. This technique could quickly degrade and remove the pollutants from a soil at relatively low temperatures. With traditional extraction techniques generating a toxic stream of waste as pollutants are removed from the solid matrix, the benefit of this technique is that it has the potential to generate clean extracts. Also, no harmful and expensive organic solvents are used in this process, and the replacement of the soil to its original site is feasible with this method because the humus properties of the soil should be preserved. While further studies are still necessary, the proposed remediation method has shown great promise in the studies performed thus far. This method has the potential to serve as an environmentally sound and efficient technique that can remove persistent aliphatic pollutants from the sediment, sludge, or soil matrix in a disturbed ecosystem.

## Figures and Tables

**Table 1:** Dieldrin degradation and extraction.

	150 °C		200 °C		250 °C	
	% Removed (%RSD)	% Degraded (%RSD)	% Removed (%RSD)	% Degraded (%RSD)	% Removed (%RSD)	% Degraded (%RSD)
100% H_2_O	80.3(178)	46.9(29)	97.8(0)	64.9(24)	99.9(0)	97.5(4)
3% H_2_O_2_	68.5(21)	41.6(23)	97.9(0)	86.8(5)	100(0)	99.4(1)

**Table 2:** Mirex degradation and extraction at 200 °C.

	200 °C		250 °C	
	% Removed (%RSD)	% Degraded (%RSD)	% Removed (%RSD)	% Degraded (%RSD)
100% H_2_O	99.0(0)	33.3(10)	99.9(0)	58.3(3)
3% H_2_O_2_	90.8(18)	50.0(40)	100(0)	58.2(17)

**Table 3:** Efficiency of DDD degradation and extraction from sand matrices.

	200 °C		250 °C	
	% Removed (%RSD)	% Degraded (%RSD)	% Removed (%RSD)	% Degraded (%RSD)
100% H_2_O	99.6(1)	51.2(7)	99.8(1)	92.6(8)
3% H_2_O_2_	84.6(16)	72.4(16)	99.8(1)	92.8(12)

**Table 4:** Efficiency of DDT degradation and extraction from an environmental solid sample.

200 °C		250 °C	
% Removed (%RSD)	% Degraded (%RSD)	% Removed (%RSD)	% Degraded (%RSD)
97.8(0.7)	94.0(0.7)	99.9(0.1)	99.8(0.1)

## Data Availability

None.

## Sample Availability

Not available.
